# Antenatal care and caesarean sections: trends and inequalities in four population-based birth cohorts in Pelotas, Brazil, 1982–2015

**DOI:** 10.1093/ije/dyy211

**Published:** 2019-03-18

**Authors:** Aluisio J D Barros, Cesar G Victora, Bernardo L Horta, Fernando C Wehrmeister, Diego Bassani, Mariângela F Silveira, Leonardo P Santos, Cauane Blumenberg, Fernando C Barros, Pelotas Cohorts Study Group, Alicia Matijasevich, Ana M B Menezes, Andrea Dâmaso Bertoldi, Helen Gonçalves, Iná S Santos, Joseph Murray, Luciana Tovo-Rodrigues, Maria Cecilia F Assunção, Marlos Rodrigues Domingues, Pedro R C Hallal

**Affiliations:** 1 University of São Paulo, Brazil; 2 Federal University of Pelotas, Brazil; 1 Postgraduate Program in Epidemiology, Federal University of Pelotas, Pelotas Brazil; 2 Centre for Global Child Health, University of Toronto, Toronto, ON, Canada; 3 Maternal and Child Health Department and Postgraduate Program in Epidemiology, Federal University of Pelotas, Pelotas, Brazil; 4 Postgraduate Program in Health and Behavior, Catholic University of Pelotas, Pelotas, Brazil

**Keywords:** Prenatal care, caesarean section, healthcare disparities, cohort studies

## Abstract

**Background:**

Antenatal care and correctly indicated caesarean section can positively impact on health outcomes of the mother and newborn. Our objective was to describe how coverage and inequalities for these interventions changed from 1982 to 2015 in Pelotas, Brazil.

**Methods:**

Using perinatal data from the 1982, 1993, 2004 and 2015 Pelotas birth cohorts, we assessed antenatal care coverage and caesarean section rates over time. Antenatal care indicators included the median number of visits, the prevalence of mothers attending at least six visits and the proportion who started antenatal care in the first trimester of pregnancy and attended at least six visits. We described these outcomes according to income quintiles and maternal skin colour, to identify inequalities. We described overall, private sector and public sector caesarean section rates. Differences in prevalence were tested using chi-square testing and median differences using Kruskal-Wallis testing.

**Results:**

From 1982 to 2015, the median number of antenatal care visits and the prevalence of mothers attending at least six visits increased in all income quintiles and skin colour groups. Inequalities were reduced, but not eliminated. The overall proportion of caesarean births increased from 27.6% in 1982 to 65.1% in 2015, when 93.9% of the births in the private sector were by caesarean section. Absolute income-related inequalities in caesarean sections increased over time.

**Conclusions:**

Special attention should be given to the antenatal care of poor and Black women in order to reduce inequalities. The explosive increase in caesarean sections requires radical changes in delivery care policies, in order to reverse the current trend.

**Key Messages**

- The number of antenatal care visits and the coverage of antenatal care indicators increased in all income quintiles and skin colour groups; however, inequalities are still sizeable.
- There was a marked increase, from 41% in 1982 to 63% in 2015, in the proportion of women with eight or more antenatal visits, which is the current recommendation by the World Health Organization.
- Regarding coverage with six or more antenatal care visits, the gap between women in the richest and poorest quintiles fell from 40% to 16% points between 1982 and 2015.
- Absolute inequalities in caesarean sections—expressed by the difference between the richest and poorest quintiles—increased over time, whereas relative inequalities—expressed as the corresponding ratio—decreased.
- The prevalence of caesarean sections is unacceptably high, being almost universal in the private sector in 2004 and 2015. Radical policies are needed to reverse the observed trend.

## Introduction

In 2015, the United Nations proposed a new agenda containing 17 goals to improve the lives of people—the Sustainable Development Goals [SDGs]—to be achieved by 2030.^1^ Goal 3 reads ‘ensure healthy lives and promote well-being for all people at all ages’,^2^ and the main specific targets aim to reduce maternal mortality, end preventable deaths of children under 5 years of age and ensure universal coverage of health services. The focus on fighting inequalities is implicit in the ‘for all people at all ages’ qualifier; unlike the Millennium Development Goals, equity is central to the SDGs.

Antenatal care [ANC] carries an essential set of interventions and actions aimed at reducing maternal and child morbidity and mortality. Quality ANC reduces the risk of maternal morbidity and mortality, and also promotes women’s health through the provision of information about risk behaviours and promotion of breastfeeding and contraception.^3^ Additionally, ANC can also prevent morbidity and mortality in children through preventive interventions [such as tetanus immunization] and early detection of problems.^4^ However, inequalities in the coverage of ANC, most often with lower coverage among the poorer and more vulnerable women, may hinder the impact of ANC on the population as a whole.^5^^,^^6^

Delivery by caesarean section is also associated with maternal and newborn survival, as it can be a lifesaving intervention when suitably indicated.^7^ In recent decades, however, caesarean section rates have increased in many countries,^8^^,^^9^ becoming more common than vaginal births in Brazil.^10^ When a large number of unnecessary caesarian deliveries are performed, the risks of complication become a concern. A large study on the risks and benefits of caesarean section has concluded that although benefiting deliveries with a breech presentation, the risk of severe maternal and neonatal morbidity and mortality for cephalic presentations was greater than for vaginal delivery. Recent epidemiological studies have linked caesarean section birth with increased risk of several outcomes later in life, such as type 1 diabetes,^11^ asthma^12^^,^^13^ and obesity,^13–15^ although the literature is not fully consistent on such associations. Nevertheless, given the possibility of scheduling the delivery [convenient to doctors and to mothers], the absence of pain during delivery and a perception of safety and technological sophistication, caesarean sections are usually much more common among mothers who are richer or private sector clients.^8^

Since 1982, four population-based birth cohorts have been started in Pelotas, a Southern Brazilian city. The 1982,^16^ 1993,^17^ 2004^18^^,^^19^ and 2015^20^ Pelotas Birth Cohort studies recruited nearly 20 000 individuals, of whom over 15 000 are still being followed up. The study covers a wide range of topics, including morbidity and mortality, growth, development and cognition, and violence. ANC and type of delivery have been extensively studied. Because the cohorts span over 33 years, it is possible to present a broad view of what happened in the country during these years, when Brazil faced significant changes in its health system and in its economy. Using data from four population-based cohort studies, we describe how ANC coverage and proportion of caesarean section among mothers changed over time. We also address changes in inequalities in terms of income and skin colour, according to the number of ANC visits and the frequency of caesarean section.

## Methods

Pelotas is a city located in the south of Brazil, with approximately 340 000 inhabitants as of 2015.^21^ The Pelotas Birth Cohorts are multipurpose longitudinal studies that follow very similar methodologies. All liveborns from mothers who lived in the urban area of the city, in the years of 1982,^16^ 1993^17^ 2004^18^^,^^19^ and 2015^20^ were recruited. All maternity hospitals in Pelotas were visited daily during each year in order to invite the mothers to take part in the studies. In the perinatal assessment, mothers were interviewed within 24 h of delivery, and the newborns were examined. The four Pelotas Birth Cohorts enrolled 6011 newborns in 1982, 5304 in 1993, 4287 in 2004 and 4329 in 2015. The recruitment was limited to maternity hospitals since they account for at least 99% of the births. The present analyses focus on the information collected in the perinatal studies. More details about the individual studies can be found in the cohort profiles, previously published.^16^^,^^17^^,^^19–21^

In the perinatal studies, trained interviewers asked each mother about the pregnancy and birth, including the total number of antenatal care visits and the month of pregnancy in which the first antenatal care visit occurred. In 1982, information on the date of the first ANC visit was only asked for women giving birth from September to December. In 2015, this variable was extracted from the antenatal care cards presented by the women. In addition, mothers were also asked about the type of delivery [vaginal or caesarean] and whether the delivery was funded by the public health system—Sistema Único de Saúde [public sector] —or through a private health insurance or out-of-pocket payment [private sector].

Four ANC indicators were studied: the median number of ANC visits, proportion of mothers attending at least six ANC visits [the proposed minimum by the Brazilian Ministry of Health for the cohort years], antenatal care adequacy according to the Kessner criteria and tproportion of mothers who had at least six ANC visits, starting in the first trimester of pregnancy, as a proxy indicator of ANC quality. Based on this indicator, the proportion of women starting ANC in the first trimester of gestation was lower in 2015 than in 2004 or 1993, and upon closer scrutiny it became evident that health care workers were routinely recording the date of the second visit—when women were returning to the clinic with laboratory examinations—rather than the first visit when the examinations had been ordered by the doctor. Thus, because of poor comparability, this last indicator was not presented for the 2015 cohort. This problem did not affect the information on the total number of visits, which was reported by women during the perinatal interview. The Kessner method classifies antenatal care as adequate when the number of visits is acceptable in relation to gestational age at birth; this method was designed to allow for the fact that shorter gestations are likely to be associated with fewer visits to a provider.^22^ This classification is more demanding than the indicator of women with six or more visits; for example, nine or more visits are required to be considered adequate when gestational age equals 36 or more weeks. Finally, we also assessed the proportion of caesarean births in each year.

Two stratifiers were used to analyse inequalities: maternal skin colour and household income. Skin colour was categorized into Black, Brown or White [except for 1982, when it was recorded just as White or non-White]. Assessment was made by the interviewers in 1982 and 1993 and based on self-report in 2004 and 2015. Due to the high level of miscegenation in the Brazilian population, it makes more sense to refer to skin colour rather than ethnicity, as is common in other countries. According to the 2010 census, carried out by the Brazilian Institute for Geography and Statistics [IBGE], 43% of the Brazilian population self-classified as ‘pardos’ [referred to as Brown in this article], mostly an admixture of African and European descendants. National censuses in Brazil have long used this skin colour classification instead of other categorizations. Household income was calculated by summing the income of all household members, and subsequently dividing total income into quintiles.

Differences in the median of ANC visits according to quintiles of household income and maternal skin colour in the four birth cohorts were assessed using the Kruskal-Wallis test. In addition, we used chi-square tests to identify differences in the proportion of caesarean sections and ANC coverage between the four birth cohorts, according to quintiles of household income and maternal skin colour and also according to public or private health sector care [caesarean sections only].

The concentration index and the slope index of inequality were calculated to assess inequalities in ANC visits and caesarean sections according to income, from 1982 to 2015. The concentration index is a relative measure of inequality and uses a similar approach to the Gini index, ordering individuals according to income on the x-axis and plotting ANC visits or caesarean sections on the y-axis. The slope index is an absolute measure of inequality obtained here, using a logistic regression of ANC visits or caesarean sections and income. More details about the use and interpretation of these indices are presented elsewhere.^23^ All analyses were performed using the Stata 15.1 software.^24^

## Results

Our analyses are based on women with information about the number of antenatal care visits and type of delivery. These amounted to 5983, 5292, 4106 and 4286 mothers from the 1982, 1993, 2004 and 2015 cohorts, respectively.

Table 1 shows that although the median number of antenatal care visits was similar in the four cohort studies, the proportion of women with eight or more visits increased considerably from around 40% in 1982 to over 60% in 2015. In 1982, 34.9% of women who started ANC in the first trimester of pregnancy had eight or more ANC visits, increasing to 47% in 1993 and 50.5% in 2004. As mentioned, the information for 2015 was not comparable and is not presented. A more detailed description of the sociodemographic characteristics of the cohort participants is presented in the methodological paper that is part of this issue.^21^ The caesarean section rate increased by almost 40% age points from 1982 to 2015, being more prevalent than vaginal deliveries in 2015. In 1982 and 1993, around 30% of women gave birth by caesarean section. In 2004 almost 50% of deliveries were by caesarean section, and 65% of women from the 2015 cohort gave birth by caesarean section [Table 1].

**Table 1.** Median number of antenatal care visits and proportion of women who attended at least 6 antenatal care visits during pregnancy, who attended at least six antenatal visits during pregnancy starting in the first trimester, and who delivered through a caesarean section, for each of the four Pelotas Birth Cohorts [1982, 1993, 2004 and 2015]

Outcomes	Birth cohort year				*P*-value^b^
	1982	1993	2004	2015	
Total number of deliveries	*n* [%]	*n* [%]	*n* [%]	*n* [%]	0.055
Live-born	5914 [98.4]	5249 [99.0]	4231 [98.7]	4275 [98.8]	
Stillbirths	97 [1.6]	55 [1.0]	56 [1.3]	54 [1.2]	
Total	6011 [100.0]	5304 [100.0]	4287 [100.0]	4329 [100.0]	
	Median [IQR]	Median [IQR]	Median [IQR]	Median [IQR]	
Median number of ANC visits	7 [5–9]	8 [5–10]	8 [6–10]	8 [6–10]	<0.001
Total number of ANC visits	*n* [%]	*n* [%]	*n* [%]	*n* [%]	<0.001
0 visits	306 [5.1]	257 [4.9]	81 [2.0]	88 [2.1]	
1-3	657 [11.0]	364 [6.9]	236 [5.8]	170 [4.0]	
4-5	1021 [17.1]	755 [14.3]	488 [11.9]	440 [10.3]	
6-7	1525 [25.5]	1205 [22.8]	927 [22.6]	905 [21.1]	
8+ visits	2474 [41.4]	2711 [51.2]	2374 [57.8]	2683 [62.6]	
Total	5983 [100.0]	5292 [100.0]	4106 [100.0]	4286 [100.0]	
Number of ANC visits starting in the 1st trimester					<0.001
Did not start in the 1sttrimester	1010 [47.3]	1626 [31.3]	1207 [29.5]	−	
1-3	21 [1.0]^a^	41 [0.8]	35 [0.9]	−	
4-5	73 [3.4]^a^	224 [4.3]	170 [4.2]	−	
6-7	285 [13.4]^a^	864 [16.6]	611 [14.9]	−	
8+	743 [34.9]^a^	2447 [47.0]	2073 [50.5]	−	
Total	2132 [100.0]^a^	5202 [100.0]	4096 [100.0]	−	
Type of delivery					<0.001
C-section	1659 [27.6]	1620 [30.5]	1937 [45.2]	2808 [64.9]	
Vaginal	4352 [72.4]	3684 [69.5]	2350 [54.8]	1520 [35.1]	
Total	6011 [100.0]	5304 [100.0]	4287 [100.0]	4328 [100.0]	
Parity					<0.001
0	2322 [39.3]	1843 [35.1]	1666 [39.3]	2112 [49.4]	
1	1661 [28.1]	1457 [27.8]	1111 [26.3]	1321 [30.9]	
2+	1929 [32.6]	1949 [37.1]	1453 [34.4]	840 [19.7]	
Total	5912 [100.0]	5249 [100.0]	4230 [100.0]	4273 [100.0]	
Maternal age					<0.001
12-19	912 [15.4]	915 [17.4]	800 [18.9]	622 [14.6]	
20-24	1843 [31.2]	1447 [27.6]	1149 [27.2]	1011 [23.6]	
25-29	1599 [27.0]	1353 [25.8]	959 [22.7]	1006 [23.5]	
30-34	973 [16.5]	956 [18.2]	758 [17.9]	1003 [23.5]	
35+	586 [9.9]	577 [11.0]	563 [13.3]	632 [14.8]	
Total	5913 [100.0]	5248 [100.0]	4229 [100.0]	4274 [100.0]	

IQR, interquartile range.

a Missing approximately three-quarters of the information regarding the starting month of antenatal care visits.

b Chi-square tests comparing the four cohorts.

Table 2 shows that the proportion of mothers with at least six ANC visits increased in all income quintiles. However, the proportion of mothers who had at least six visits was always higher and almost universal in women in the richest group in 2004 and 2015. The prevalence of at least six antenatal care visits was also higher in White mothers, compared with Brown and Black mothers, in all four cohorts. The median numbers of visits by income and skin colour are presented in Supplementary Table 1, available as Supplementary data at *IJE* online, and show patterns similar to those observed in Table 2.

**Table 2.** Proportion of mothers who attended at least six antenatal visits during pregnancy, according to quintiles of family income and skin colour, for each of the four Pelotas Birth Cohorts [1982, 1993, 2004 and 2015]

Variable	Birth cohort year				*P*-value
	1982	1993	2004	2015	
	% [CI 95%]	% [CI 95%]	% [CI 95%]	% [CI 95%]	
Total	3999 [66.8]	3916 [74.0]	3301 [80.4]	3588 [83.7]	
Quintiles of family income					
Q1 [poorest]	44.9 [42.; 47.7]	58.9 [55.9;- 61.9]	66.8 [63.6; 70.0]	70.0 [66.9; 73.1]	<0.001
Q2	59.1 [56.3; 61.8]	68.1 [65.5; 70.8]	71.0 [67.9; 74.1]	78.7 [76.0; 81.5]	<0.001
Q3	68.0 [65.4; 70.7]	75.9 [73.1; 78.7]	80.9 [78.1; 83.6]	85.6 [83.2; 88.0]	<0.001
Q4	77.6 [75.3; 80.0]	79.1 [76.6; 81.6]	89.0 [86.8; 90.6]	88.4 [86.3; 91.1]	<0.001
Q5 [richest]	85.3 [83.3; 87.3]	90.3 [88.4; 92.1]	94.8 [93.3; 96.3]	95.7 [94.3; 97.1]	<0.001
Skin colour					
White	69.9 [68.6; 71.2]	78.1 [76.9; 79.4]	83.8 [82.4; 85.1]	86.9 [85.7; 88.1]	<0.001
Brown	−^a^	63.7 [57.5; 69.9]	78.3 [73.5; 83.1]	78.1 [74.7; 81.5]	<0.001
Black	52.8 [49.8; 55.8]	59.1 [56.0; 62.2]	68.8 [65.6; 72.0]	73.9 [70.5; 77.3]	<0.001

CI – Confidence interval.

a Absent category in the 1982 birth cohort. In 1982, mother’s skin colour was recorded as White or Other.

Results for antenatal care adequacy according to the Kessner classification are presented in Supplementary Table 2, available as Supplementary data at *IJE* online. Given that this is a more stringent classification than the previous indicator on six or more visits, it is not surprising that coverage levels are lower for all income and skin colour groups, and that inequalities become even more evident. A similar situation was observed when we assessed the proportion of mothers who had started ANC visits in the first trimester of pregnancy and attended at least six ANC visits. The proportions of women increased with time, being higher among high-income and White mothers [Table 3].

**Table 3.** Proportion of mothers who attended at least six antenatal visits during pregnancy starting in the first trimester, according to quintiles of family income and skin colour, for each of the four Pelotas Birth Cohorts [1982, 1993, 2004 and 2015]

Variable	Birth cohort year				*P*-value
	1982^a^	1993	2004	2015	
	% [CI 95%]	% [CI 95%]	% [CI 95%]	% [CI 95%]	
Total	1028 [48.2]	3311 [63.7]	2684 [65.5]	−	<0.001
Quintiles of family income					
Q1 [poorest]	32.3 [27.6; 37.4]	51.0 [47.8; 54.3]	53.5 [49.7; 56.7]	−	<0.001
Q2	45.8 [40.6; 51.2]	60.6 [57.7; 63.5]	52.1 [48.7; 55.6]	−	<0.001
Q3	54.0 [48.7; 59.2]	67.0 [63.8; 70.1]	65.8 [62.3; 69.0]	−	<0.001
Q4	62.9 [58.0; 67.6]	71.8 [68.9; 74.5]	77.9 [74.9; 80.6]	−	<0.001
Q5 [richest]	81.7 [77.6; 85.2]	84.7 [82.3; 86.8]	87.8 [85.3; 89.8]	−	<0.001
Skin colour					
White	59.2 [56.7; 61.7]	70.8 [69.3; 72.2]	71.7 [70.0; 73.3]	−	<0.001
Brown	−^b^	57.1 [50.3; 63.7]	63.9 [58.1; 69.4]	−	<0.001
Black	41.8 [36.4; 47.5]	52.6 [49.3; 56.0]	53.0 [49.4; 56.5]	−	< 0.001

CI, confidence interval.

a The information regarding the starting month of antenatal care visits is missing for approximately three-quarters of the mothers.

b Absent category in the 1982 birth cohort. In 1982, mother’s skin colour was recorded as White or Other.

**Figure 1.**
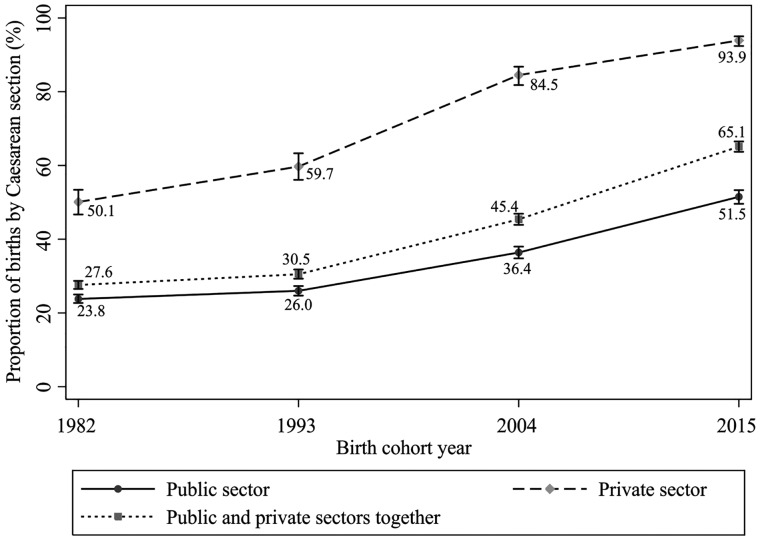
Proportion of births by Caesarean section, total and by public or private sector, for each Pelotas birth cohort (1982, 1993, 2004 and 2015). The 95% confidence intervals are indicated by the vertical lines for each data point.

The prevalence of caesarean sections increased from 1982 to 2015 for all income quintiles [Table 4], but remained highest in the richest quintile, among whom nearly 90% gave birth by caesarean section in 2015. Caesarean sections were more common than vaginal deliveries in the four cohort studies when delivery was paid by private health insurance or out of pocket. In 2004 and 2015, caesarean sections were almost universal in the private sector [84.5% and 93.9%, respectively]. There was also a marked increase in caesarean sections in the public sector from 1982 to 2015, from 24% to over 50% of all deliveries [Figure 1].

**Table 4.** Proportion of births by caesarean section according to quintiles of family income, for the four Pelotas Birth Cohorts [1982, 1993, 2004 and 2015]

Variable	Birth cohort year				*P*-value
	1982	1993	2004	2015	
	*n* [%]	*n* [%]	*n* [%]	*n* [%]	
Total	1659 [27.6]	1620 [30.5]	1937 [45.2]	2808 [64.9]	<0.001
	% [CI 95%]	% [CI 95%]	% [CI 95%]	% [CI 95%]	
Quintiles of family income					
Q1 [poorest]	16.8 [14.7; 18.9]	23.6 [21.0; 26.1]	38.3 [35.1; 41.5]	50.5 [47.1; 53.8]	<0.001
Q2	24.4 [22.0; 26.8]	23.1 [20.7; 25.5]	35.1 [31.9; 38.3]	55.4 [52.1; 58.7]	<0.001
Q3	22.0 [19.6; 24.3]	27.4 [24.5; 30.3]	42.2 [38.9; 45.6]	63.7 [60.5; 66.9]	<0.001
Q4	31.8 [29.1; 34.4]	32.9 [30.0; 35.8]	46.6 [43.3; 49.9]	68.7 [65.6; 71.8]	<0.001
Q5 [richest]	43.4 [40.5; 46.2]	47.0 [44.0; 50.1]	64.5 [61.2; 67.7]	86.2 [83.9; 88.5]	<0.001
Skin colour					
White	28.9 [27.7; 30.2]	32.1 [30.7; 33.6]	46.3 [44.6; 48.1]	68.0 [66.4; 69.6]	<0.001
Brown	−^a^	27.4 [22.1; 33.5]	45.2 [39.6; 50.9]	57.6 [53.5; 61.7]	<0.001
Black	21.6 [19.3; 24.2]	24.8 [22.2; 27.6]	41.1 [37.9; 44.4]	56.3 [52.5; 60.1]	<0.001

CI, confidence interval.

a Absent category in the 1982 birth cohort. In 1982, mother’s skin colour was recorded as White or Other.

Absolute income-related inequality decreased over time for more than six ANC visits. The slope index dropped 17% age points from 1982 to 2015, but most of the reduction was concentrated between 1982 and 1993. From 2004 to 2015, the reduction was only 6% age points. For more than six ANC visits starting in the first trimester, the pattern was less clear. There was a 17-percentage point reduction in absolute inequality from 1982 to 1993, but it increased in 2004 by 7% age points. Relative inequality followed approximately the same pattern [Table 5]. Sizeable inequalities persist in 2015.

**Table 5.** Income inequalities for attending at least six antenatal care visits, for attending at least six antenatal care visits starting in the first trimester of pregnancy and for caesarean section

Outcome	Birth cohort year			
	1982	1993	2004	2015
At least six antenatal visits				
Slope index of inequality [SII]	47.3 [43.9; 50.8]	36.0 [32.2; 39.8]	36.5 [32.6; 40.5]	30.3 [26.5; 34.1]
Concentration index [CIX]	11.5 [10.5; 12.6]	4.5 [3.6; 5.5]	5.3 [4.4; 6.1]	4.2 [3.5; 4.9]
At least six antenatal visits starting in the first trimester of pregnancy				
Slope index of inequality [SII]	55.0 [49.0; 60.9]	37.9 [33.8; 42.0]	45.0 [40.7; 49.3]	−
Concentration index [CIX]	10.0 [7.7; 12.4]	4.0 [2.9; 5.2]	6.1 [4.9; 7.4]	−
Caesarean section				
Slope index of inequality [SII]	29.3 [25.6; 33.1]	27.6 [23.3; 31.8]	30.5 [25.7; 35.3]	40.6 [36.3; 44.8]
Concentration index [CIX]	17.1 [14.7; 19.4]	13.6 [11.2; 16.0]	9.7 [7.8; 11.6]	10.3 [9.1; 11.6]

With a steep overall increase in caesarean sections, especially for the richest mothers, it is not surprising that we see an increase in absolute inequality along time. The slope index increased from around 29% to nearly 41% age points from 1982 to 2015. The increase in inequality was more marked in the last period. For relative inequality the trend was the opposite, with a modest reduction in the concentration index from 17 to 10.

## Discussion

Our study is based on four population-based birth cohorts from Pelotas [Brazil]. Despite not being nationally representative by design, these studies reflect the national trends in the country, given that the Brazilian public health system has national coverage and is largely funded and regulated by the federal government. Further, despite being geographically distant, Pelotas is a medium-sized city by Brazilian standards, with a per capita GDP close to the municipalities average; Pelotas has a per capita GDP of R$21 553, compared with a national average of R$19 504 [2015 estimates, source: IBGE URL: https://goo.gl/f6kgN2]. We explored antenatal care and caesarean sections, two important aspects of maternal health. ANC presented remarkable improvements on all income quintiles from 1982 to 2015. Income inequalities, in both absolute and relative terms, were markedly reduced. At the same time, the proportion of deliveries by caesarean section increased so markedly that in 2015 caesarean sections were more common than vaginal deliveries, in both private and public sectors.

The high prevalence of caesarean sections is not restricted to Pelotas, but was recorded in Brazil as a whole. A recent global analysis, comparing 150 countries, showed that in 2014 Brazil was second only to the Dominican Republic, with 55.6% and 56.4% caesarean deliveries, respectively.^25^ An assessment of caesarean sections in Brazil concluded that they were more common among women at low risk of maternal or fetal death, suggesting that the option for this type of delivery was mostly elective.^26^ A study performed in the Brazilian state of Rio de Janeiro not only supports this hypothesis but places a good deal of responsibility on the health services, since they showed that 70% of the women did not report a preference for caesarean section at the start of pregnancy, but in the end 90% underwent caesarean section.^27^ A national study found a similar result for primiparous women in the private sector, but a less extreme change in the public sector. Here, 72% of the women declared a preference for a vaginal delivery, and in the end 43% delivered vaginally. The reasons behind the preference for deliveries by caesarean section are many, involving women’s fear of pain,^27^^,^^28^ financial benefits for the hospitals or doctors,^29^ ability to schedule the delivery on a given day and the idea that caesarean sections are related to better quality care since they are preferred by rich women.^26^ Women who declared a preference for a vaginal delivery mostly referred to a better recovery after a normal birth.^28^ In our study, caesarean section rates were unacceptably high, mainly among the more affluent women or those who gave birth in private sector care. The World Health Organization [WHO] has recently re_asserted its position on the ideal proportion of caesarean sections, and showed that values above 10% are not associated with reduced mortality outcomes.^30^

Inequalities in caesarean section rates behaved in a way that may seem odd, with absolute inequality increasing and relative inequality decreasing. However, with a steep increase in overall rates this is not an uncommon pattern, given that ratios tend to decline when the coverage for all groups increase. What is clear is that the distance between the extremes of the wealth distribution increased in the period between 2004 and 2015, with an increase of 21% age points in caesarean section rates for the richest group, and an increase of 12%age points for the poorest group.

In contrast, the increase in antenatal care coverage from 1982 to 2015 is an important step towards achieving universal health coverage, as proposed by SDG number 3.^2^ This increase occurred in all income quintiles. In 2015, even before the new World Health Organization guidelines recommended at least eight antenatal visits, 63% of the women had already achieved this goal.^31^ ANC coverage in Brazil, regardless of the indicator used, is substantially higher and wealth-related inequalities are considerably less marked than in most other low- and middle-income countries.^32^ Nevertheless, in spite the observed reduction in both absolute and relative inequalities, our results still reveal marked differences in ANC coverage across income and ethnic groups. In 2015, the slope index still showed a 30-percentage point difference between the extremes of income distribution, and there was a 13-percentage point difference between White and Black mothers for more than six ANC visits. The observed patterns of inequality are confirmed by our analyses using the Kessner index for antenatal care [Supplementary Table 2, available as Supplementary data at *IJE* online], which accounts for the fact that shorter durations of gestation tend to be associated with fewer visits.^22^ Data from national surveys confirm the pattern of decreasing inequalities. These analyses show that whereas coverage with at least one ANC visit was almost universal in the whole country, there were important inequalities in coverage with four visits, and even greater disparities when six visits were considered.^33^

Our results suggest that ANC coverage is consistently lower in those groups of women that typically present higher risks of maternal and infant mortality.^3^^,^^4^ To achieve the SDG goal of ‘ensure healthy lives and promote well-being for all people at all ages’, it is essential to achieve higher coverage in the most vulnerable groups. In Brazil, home ANC is already part of the Family Health Strategy targeted at vulnerable communities throughout the country, which may explain the decline in inequality.^34^

The Pelotas Birth Cohorts are rigorous studies that share location, methods and recruitment strategy, and thus enable us to draw a very precise picture of trend in antenatal and delivery care over more than 30 years. Nevertheless, some limitations must be noted. In 1982, information on the date of the first ANC visit was not collected for deliveries taking place from January to August. In this cohort, skin colour was coded as White or Other, instead of the three categories [White, Brown And Black] used in the later studies. The colour distribution for the four cohorts suggests that most women classified as Brown women ended up classified as White. Another important limitation was the lack of comparability between the 2015 and the earlier cohorts regarding the gestational age at the first ANC visit, which yielded lower estimates for the indicator of six or more visits starting in the first trimester, and also resulted in a larger percentage of missing information [around 12%]. In all cohorts, most of the information was based on the mother’s report in the perinatal interview, and there is always the risk of recall bias, especially considering that the hours after delivery are a time when a recent mother is mostly focused on the newborn. Finally, the timing of the first antenatal visit is particularly challenging for some of the mothers.

Our results showed an increase in the number of antenatal care visits in all income quintiles and skin colour groups, as well as an increase in ANC indicators coverage. In spite of this, inequalities are still sizeable. Special attention should be given to poor and Black women in order to increase their access to ANC and reduce these inequalities. We also showed that the prevalence of caesarean section is unacceptably high, especially among the women who are richer or have deliveries in the private sector. Reversing the current trend requires radical changes in delivery care policies.

## Funding

The four cohorts received funding from the following agencies: the Wellcome Trust [UK], International Development Research Center [IDRC, Canada], World Health Organization [WHO, Geneva], Overseas Development Administration [ODA, UK], European Union, Brazilian National Support Program for Centers of Excellence [PRONEX, Brazil], Brazilian National Council for Scientific and Technological Development [CNPq, Brazil], Science and Technology Department [DECIT] of the Brazilian Ministry of Health [Brazil], Research Support Foundation of the State of Rio Grande do Sul [FAPERGS, Brazil], Brazilian Child's Pastorate [Brazil] and Brazilian Collective Health Association Health [ABRASCO, Brazil].

## Supplementary Material

Supplementary Tables

## Pelotas Cohorts Study Group

Alicia Matijasevich,^1^ Ana M B Menezes,^2^ Andrea Dâmaso Bertoldi,^2^ Helen Gonçalves,^2^ Iná S Santos,^2^ Joseph Murray,^2^ Luciana Tovo-Rodrigues,^2^ Maria Cecilia F Assunção,^2^ Marlos Rodrigues Domingues^2^ and Pedro R C Hallal.^2^

^1^University of São Paulo, Brazil and ^2^Federal University of Pelotas, Brazil.
