# Supplementary material for: Antenatal care and caesarean sections: trends and inequalities in four population-based birth cohorts in Pelotas, Brazil, 1982–2015
Source: Int J Epidemiol. 2019 Mar 18;48(Suppl 1):i37–45. doi: 10.1093/ije/dyy211 (PMC6422067; doi:10.1093/ije/dyy211)
Supplement: Supplementary Tables [file dyy211_supplementary_tables.docx]

**SUPPLEMENTARY INFORMATION**

| **Supplementary table 1.** Median number of antenatal visits according to quintiles of family income and mother skin color per birth cohort year. | | | | | | | | | |
| --- | --- | --- | --- | --- | --- | --- | --- | --- | --- |
| **Variable** | **Birth cohort year** | | | | | | |  | **p-value** |
|  | **1982** |  | **1993** |  | **2004** |  | **2015** |  |  |
| Quintiles of family income | Median (IQR) |  | Median (IQR) |  | Median (IQR) |  | Median (IQR) |  |  |
| *p-value* | *<0.001* |  | *<0.001* |  | *<0.001* |  | *<0.001* |  |  |
| Q1 (Poorest) | 5 (3 - 7) |  | 6 (4 - 8) |  | 7 (5 - 9) |  | 7 (5 - 9) |  | *< 0.001* |
| Q2 | 6 (4 - 8) |  | 7 (5 - 9) |  | 7 (5 - 9) |  | 8 (6 - 10) |  | *< 0.001* |
| Q3 | 7 (5 - 8) |  | 8 (6 - 10) |  | 8 (6 - 10) |  | 8 (7 - 10) |  | *< 0.001* |
| Q4 | 7 (6 - 9) |  | 8 (6 - 10) |  | 9 (7 - 10) |  | 9 (7 - 10) |  | *< 0.001* |
| Q5 (Richest) | 9 (7 - 10) |  | 10 (8 - 12) |  | 10 (8 - 12) |  | 10 (8 - 12) |  | *< 0.001* |
| Skin color |  |  |  |  |  |  |  |  |  |
| *p-value* | *<0.001* |  | *<0.001* |  | *<0.001* |  | *<0.001* |  |  |
| White | 7 (5 - 9) |  | 8 (6 - 10) |  | 8 (6 - 10) |  | 9 (7 - 11) |  | *< 0.001* |
| Brown^a^ | -^a^ |  | 7 (5 - 10) |  | 8 (6 - 10) |  | 8 (6 - 10) |  | *< 0.001* |
| Black | 6 (3 - 8) |  | 6 (4 - 8) |  | 7 (5 - 9) |  | 7 (5 - 10) |  | *< 0.001* |
| ^a^Absent category in the 1982 birth cohort. | | | | | | | | | |
| IQR - interquartile range | | | | | | | | | |

| **Supplementary table 2.** Proportion of mothers with adequate antenatal care according to Kessner index by quintiles of family income and skin color per birth cohort year.   \| **Variable** \| **Birth cohort year** \| \| \| \| **p-value** \| \| --- \| --- \| --- \| --- \| --- \| --- \| \| **1982** \| **1993** \| **2004** \| **2015** \| \|  \| N (%) \| N (%) \| N (%) \| N (%) \|  \| \| Total \| 1,436 (30.4) \| 2,082 (40.2) \| 1,878 (45.9) \| 2,191 (51,1) \|  \| \|  \|  \|  \|  \|  \|  \| \| Quintiles of family income \| % (CI 95%) \| % (CI 95%) \| % (CI 95%) \| % (CI 95%) \|  \| \| Q1 (Poorest) \| 14.4 (12.0; 16.8) \| 23.3 (20.7; 25.9) \| 30.9 (27.8; 34.0) \| 35.9 (32.7; 39.1) \| *<0.001* \| \| Q2 \| 20.6 (17.9; 23.2) \| 30.1 (27.5; 32.8) \| 32.3 (29.1; 35.5) \| 40.4 (37.1; 43.7) \| *<0.001* \| \| Q3 \| 24.2 (21.5; 27.0) \| 41.1 (37.9; 44.4) \| 43.6 (40.2; 47.1) \| 48.4 (45.1; 51.8) \| *<0.001* \| \| Q4 \| 32.6 (29.7; 35.5) \| 45.6 (42.5; 48.7) \| 52.1 (48.7; 55.5) \| 56.6 (53.3; 59.9) \| *<0.001* \| \| Q5 (Richest) \| 55.2 (52.2; 58.3) \| 63.9 (61.0; 66.9) \| 71.0 (67.9; 74.1) \| 74.3 (71.3; 77.2) \| *<0.001* \| \| Skin color \|  \|  \|  \|  \|  \| \| White \| 32.4 (30.9; 33.8) \| 44.2 (42.6; 45.7) \| 49.7 (48.0; 51.5) \| 55.5 (53.7; 57.2) \| *<0.001* \| \| Brown \| ---^a^ \| 33.0 (26.9; 39.2) \| 40.6 (34.9; 46.3) \| 43.5 (39.4; 47.6) \| *<0.001* \| \| Black \| 20.0 (17.2; 22.8) \| 25.2 (22.4; 28.0) \| 33.6 (30.3; 36.8) \| 37.5 (33.8; 41.3) \| *<0.001* \| \| ^a^ Absent category in the 1982 birth cohort. In 1982, mother’s skin color was recorded as white or other.  CI – Confidence interval \| \| \| \| \| \| |
| --- | --- | --- | --- | --- | --- | --- | --- | --- | --- | --- | --- | --- | --- | --- | --- | --- | --- | --- | --- | --- | --- | --- | --- | --- | --- | --- | --- | --- | --- | --- | --- | --- | --- | --- | --- | --- | --- | --- | --- | --- | --- | --- | --- | --- | --- | --- | --- | --- | --- | --- | --- | --- | --- | --- | --- | --- | --- | --- | --- | --- | --- | --- | --- | --- | --- | --- | --- | --- | --- | --- | --- | --- | --- | --- | --- | --- | --- | --- | --- | --- | --- | --- | --- | --- | --- | --- | --- | --- | --- | --- | --- | --- | --- | --- |
